# A universal model for predicting coronary artery lesions in subgroups of kawasaki disease in China: based on cluster analysis

**DOI:** 10.3389/fcvm.2025.1532768

**Published:** 2025-03-12

**Authors:** Chuxiong Gong, Feng Li, Zhongjian Su, Yanan Fu, Xing Zhang, Qinhong Li, Xiaomei Liu, Lili Deng

**Affiliations:** ^1^ Cardiovascular Department, Kunming Children’s Hospital, Kunming, Yunnan, China; ^2^ Department of Infectious Diseases, Kunming Children’s Hospital, Kunming, Yunnan, China

**Keywords:** kawasaki disease, cluster analysis, coronary artery lesions, cardiovascular diseases, children's diseases

## Abstract

**Objective:**

Coronary artery lesions (CAL) represent the most severe complication of Kawasaki disease (KD). Currently, there is no standardized method for predicting CAL in KD, and the predictive effectiveness varies among different KD patients. Therefore, our study aims to establish distinct predictive models for CAL complications based on the characteristics of different clusters.

**Methods:**

We employed principal component clustering analysis to categorize 1,795 KD patients into different clustered subgroups. We summarized the characteristics of each cluster and compared the occurrence of CAL components within each cluster. Additionally, we utilized LASSO analysis to further screen for factors associated with CAL. We then constructed CAL predictive models for each subgroup using the selected factors and conducted preliminary validation and assessment.

**Results:**

Through PCA analysis, we identified three clusters in KD. We developed predictive models for each of the three clusters. The AUCs of the three predictive models were 0.789 (95% CI: 0.732–0.845), 0.894 (95% CI: 0.856–0.932), and 0.773 (95% CI: 0.727–0.819), respectively, all demonstrating good predictive performance.

**Conclusion:**

Our study identified the existence of three clusters among KD patients. We developed KD-related CAL predictive models with good predictive performance for each cluster with distinct characteristics. This provides reference for individualized precision treatment of KD patients and aids in the health management of coronary arteries in KD.

## 1 Introduction

KD is an immune-mediated acute systemic vasculitis. Although KD can affect individuals of any age, it primarily impacts children under five years old. The incidence is typically higher in males, approximately 1.5–2 times that of females (1). KD is more prevalent in East Asia. In addition to the acute inflammatory response, some patients may experience complications (2). The most severe complication is CAL resulting from KD. CAL refers to inflammation and fibrosis of the coronary artery intima caused by KD, leading to arterial stenosis, thrombosis, and subsequent myocardial ischemia (3, 4). This damage significantly affects the cardiovascular system and overall health of children and may predispose them to cardiovascular disease in adulthood. Approximately 25% of children with KD develop CAL, which accounts for nearly all deaths in KD cases (5, 6). While the use of intravenous immunoglobulin (IVIG) has significantly reduced the incidence of CAL, some patients still experience this complication.

Currently, the diagnosis of KD primarily relies on clinical symptoms, including fever duration, conjunctival involvement, oral mucosal lesions, rash, and cervical lymphadenopathy. There are no definitive laboratory markers or imaging features to aid in early diagnosis (7, 8). This often leads to delayed diagnosis and misdiagnosis, particularly in patients with atypical clinical presentations (3). Such delays can hinder acute treatment and increase the risk of complications. In addition to CAL, complications can include IVIG resistance, liver injury, anemia, and jaundice (9–11).

Although KD is recognized as a singular disease entity, literature supports its heterogeneity. The American Heart Association (AHA) guidelines and expert consensus in China outline various clinical manifestations and systemic involvement of KD. One study analyzed transcriptomic and proteomic data, revealing that KD may present diversely due to different etiologies (12). Hao Wang et al. conducted cluster and subgroup analyses of KD patients, demonstrating significant heterogeneity in adverse outcomes, such as IVIG non-responsiveness and CAL, associated with four subgroups: liver involvement with elevated liver enzymes, high neutrophil counts, high inflammatory markers, and younger age at onset (13). Given the diversity in clinical presentation and outcomes of KD, subgroup studies are increasingly necessary. Therefore, our research aims to analyze the clinical characteristics of children with KD to identify risk factors for CAL in different subgroups, providing a theoretical basis for improving KD prognosis and outcomes.

## 2 Materials and methods

### 2.1 Participants

Our study retrospectively collected 1795 Kawasaki disease cases and relevant clinical data diagnosed at Kunming Children's Hospital from December 2014 to December 2023. This data represented an expansion of case numbers based on prior research. All cases met the American Heart Association (AHA) criteria for KD. Diagnoses of complete KD (CKD), incomplete KD (IKD), CAL, and IVIG were also performed according to AHA standards. The ethical approval was waived by the Ethics Committee of Kunming Children's Hospital (2023-05-016-K01).

#### 2.1.1 Treatment plan

For the routine treatment of Kawasaki disease, we use aspirin (30–50 mg/kg/day, divided into three doses) in combination with IVIG (2 g/kg, infused within 24 h). The dosage of aspirin is reduced to 3–5 mg/kg/day, divided into three doses, after three days of high fever. For patients who are resistant to IVIG, we administer a second dose of IVIG (2 g/kg, infused within 24 h). If fever persists, we treat with oral methylprednisolone (2 mg/kg, intravenously, twice daily). Additionally, we actively provide symptomatic treatment for patients with liver function impairment and other related conditions.

#### 2.1.2 Exclusion criteria

(1) Patients with underlying diseases such as cardiovascular diseases, liver diseases, kidney diseases, hematological disorders, immune system diseases, and hereditary metabolic endocrine disorders (2); Children with a history of KD (3); KD patients who received treatment with IVIG, aspirin, or corticosteroids prior to admission (4); KD patients with incomplete.

### 2.2 Comparison of clinical features

The data from our study were obtained from the results of examinations conducted at the time of initial hospitalization of the patients, before any treatment was administered. The variables included were 3 demographic characteristics (age of onset, sex, and race), 10 laboratory results (hemoglobin, white blood cell count, platelet count, percentage of neutrophils, percentage of lymphocytes, erythrocyte sedimentation rate (ESR), C-reactive protein (CRP), alanine aminotransferase (ALT), total bilirubin (TBIL), and gamma-glutamyl transferase (GGT)), 4 physical examination findings (oral mucosal involvement, conjunctival injection, cervical lymphadenopathy, and rash), as well as fever days (Representing the fever duration before treatment) and IVIG days (Representing the total disease course before IVIG treatment). These variables were incorporated into principal components analysis (PCA) and univariate logistic regression analysis.

The Z score refers to the maximum Z score of the left anterior descending coronary artery and the right coronary artery within 60 days after the onset of fever. The Z scores for all patients were recalculated uniformly based on the formula used for Z scores at the data collection cutoff date. The formula for calculating the Z score is based on the patient's age, height, and weight.

### 2.3 Subgroup analysis: PCA

First, the Hopkin statistic was calculated using the factoextra package to quantify the clustering tendency of the phenotypic data. Next, PCA analysis was conducted using the FactoMineR package, which included data standardization, principal component analysis, hierarchical clustering, and grouping of the hierarchical tree based on the optimal number of clusters. The optimal number of clusters was determined by comparing inertia. The PCA analysis method is similar to our previous subgroup study.

### 2.4 Analysis of risk factors and construction of predictive model

As a major complication of KD, CAL exhibited different outcomes across various subgroups. Therefore, our study first compared the clinical characteristics of the CAL and nCAL groups within each subgroup, retaining variables that showed statistically significant differences. We then conducted a Least Absolute Shrinkage and Selection Operator (LASSO) analysis using the “glmnet” package in R to identify more important predictive factors for outcomes. LASSO is a regularization method used in linear regression that increases the L1 penalty, thereby aiding in variable selection and model simplification. Its main advantage lies in reducing the coefficients of unimportant variables to zero, retaining only key variables to lower model complexity and prevent overfitting. We employed five-fold cross-validation to select the optimal lambda value, which could be lambda.min or lambda.1se, where a larger lambda signifies stronger regularization, resulting in a reduction in the number of selected variables. We filtered variables based on the best and maximum lambda values. We then calculated their Youden index and determined their cutoff values and AUC based on the Youden index. Finally, we incorporated the variables selected by the LASSO analysis into multivariable logistic regression models using the “rmda” R package to develop predictive models for each subgroup. We also developed nomograms for each subgroup using the “rms” R package to visualize the best predictive models for the respective groups.

To evaluate the predictive models for each subgroup, we first used the “pROC” R package to plot the Receiver Operating Characteristic (ROC) curve and assessed model performance using the Area Under the Curve (AUC). Subsequently, we evaluated model calibration by plotting a calibration curve with the “ResourceSelection” R package and conducted Decision Curve Analysis (DCA) using the “decision_curve” function for further accuracy assessment. Finally, we applied five-fold cross-validation using the createFolds function.

### 2.5 Statistical analysis

This study used R software (version 4.4.1) for all analyses. Continuous data with a normal distribution were described using mean ± standard deviation. Non-normally distributed data were described using median [P_25_, P_75_]. Categorical data were expressed as frequency (percentage). For continuous data, *t*-tests or rank-sum tests were used based on normality and homogeneity of variance tests. Categorical data were analyzed using chi-square tests. Risk factor screening utilized univariate logistic regression to explore influencing factors. The results of the logistic regression include the OR (odds ratio), the 95% CI (confidence interval) of the OR, and *p*. All univariate and multivariate statistical analyses were considered statistically significant at a two-sided *p* < 0.05.

## 3 Results

### 3.1 Results of PCA

**Figure 1:**
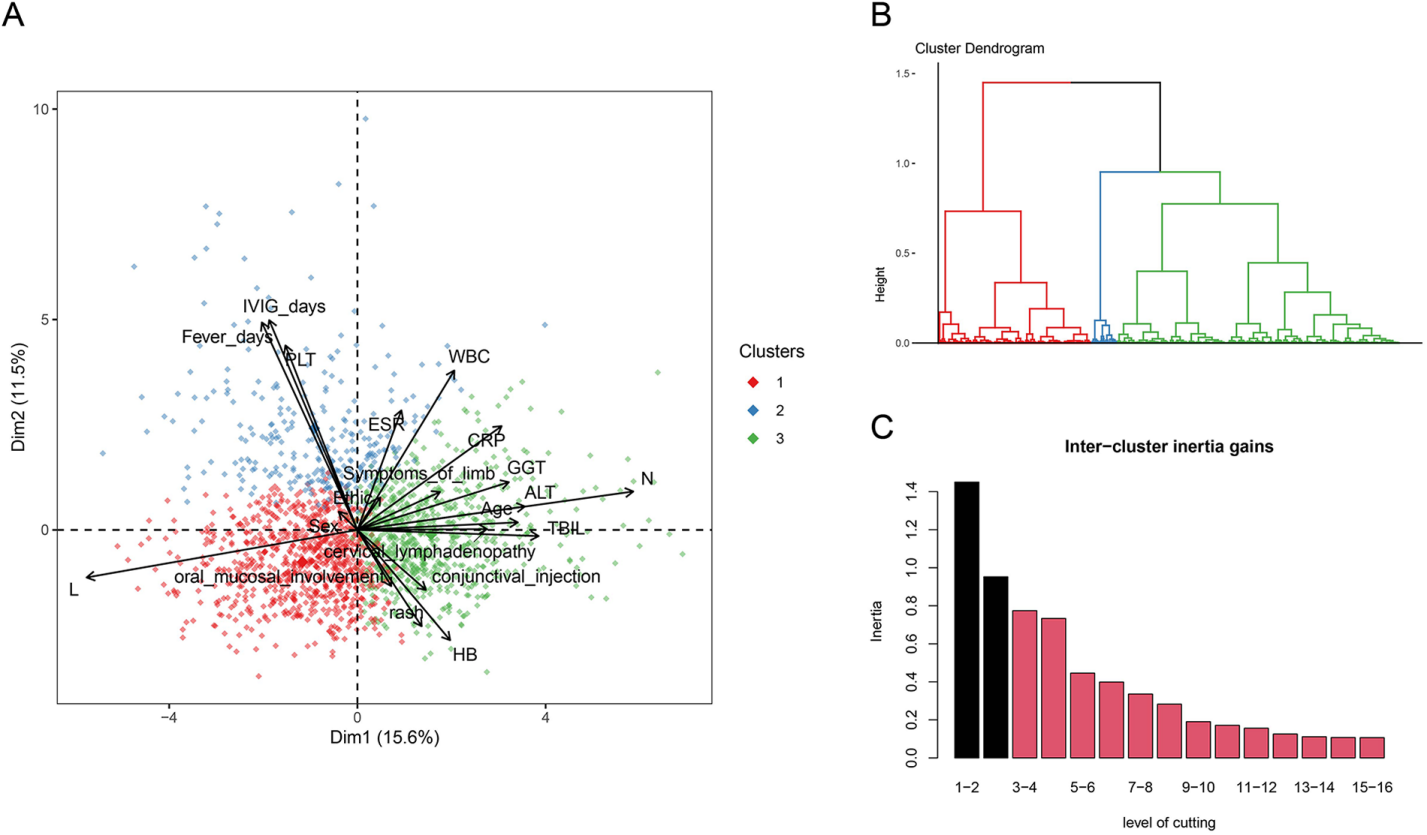
**(A)** three-dimensional clustering map of variables and loading plot of variables in PCA. **(B)** Dendrogram of the hierarchical map and identified clusters. **(C)** Calculation of the optimal number of clusters.

Our study included a total of 1,795 patients with KD. Among these, 1,235 patients (68.8%) were diagnosed with CKD, while 560 patients (31.2%) had IKD. Additionally, 388 patients (21.62%) presented with CAL, and 153 patients (8.52%) exhibited IVIG resistance. Subsequently, we performed PCA. First, we calculated the Hopkins statistic to be 0.804, indicating a significant clustering tendency within the dataset. We achieved dimensionality reduction through PCA, where principal component 1 explained 15.6% of the variance, and principal component 2 explained 11.5% of the variance (Figure 1A). Following this, we determined the optimal number of clusters to be 3 (k = 3) based on inertia calculations (Figures 1B,C). We also presented a statistical comparison of the demographic characteristics, laboratory results, and clinical features among the three identified clusters (s1).

Cluster 1 was characterized by the youngest age of the patients, the lowest levels of CRP, ESR, and WBC. It had the lowest incidence of the primary adverse outcome, CAL, and the lowest rate of IVIG resistance. Cluster 2 was marked by a high proportion of IKD, a low occurrence of typical clinical manifestations, and longer fever days and IVIG days. It had the highest incidence of the primary adverse outcome, CAL, and the highest rate of IVIG resistance. Cluster 3 was characterized by elevated ALT, GGT, and TBIL, indicating liver involvement. It had a lower incidence of the primary adverse outcome, CAL, but a higher rate of IVIG resistance.

### 3.2 Patient’s characteristics for 3 clusters

Cluster 1 included 84 cases in the CAL group and 692 cases in the nCAL group. A summary of the demographic characteristics, laboratory test results, and clinical features of the two groups was presented (s2). Among these, PLT, WBC, N, L, ESR, CRP, GGT, IVIG days and symptoms of limb showed significant differences between the two groups. Cluster 2 included 174 cases in the CAL group and 104 cases in the nCAL group. A summary of the demographic characteristics, laboratory test results, and clinical features of the two groups was presented (s3). Among these, PLT, WBC, N, L, ESR, CRP, GGT, TBIL, fever days, IVIG days, Sex, symptoms of limb and conjunctival injection showed significant differences between the two groups. Cluster 3 consisted of 130 cases in the CAL group and 611 cases in the nCAL group. A summary of the demographic characteristics, laboratory test results, and clinical features of both groups is provided (s4). Here, significant differences were noted between the two groups for PLT, WBC, ESR, CRP, TBIL, GGT, fever days, IVIG days, sex, conjunctival injection, and cervical lymphadenopathy.

### 3.3 Model development for three clusters

First, we included the statistically different variables from the comparisons between the subgroups into LASSO for variable selection. The results indicated that in Cluster 1, PLT and Symptoms of limb were selected. In Cluster 2, PLT, CRP, TBIL, IVIG days, Sex, and Symptoms of limb were selected. In Cluster 3, PLT, CRP, WBC, and IVIG days were selected.

**Figure 2:**
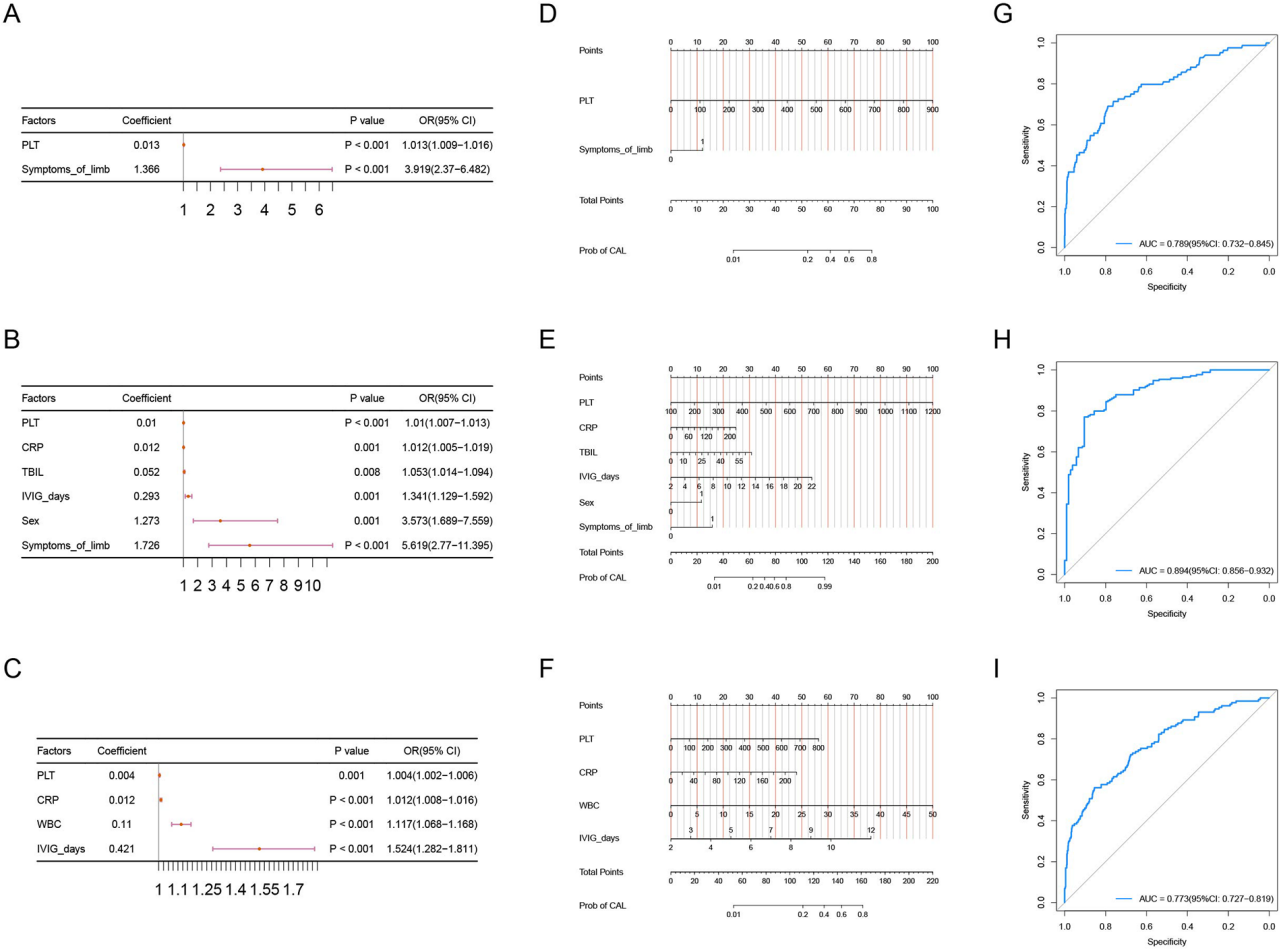
**(A**–**C)** forest plot of the results from the multinomial logistic regression analysis for cluster1, cluster2, and cluster3.

Then, we included the variables selected by LASSO into a multifactor logistic regression analysis to construct predictive models for concurrent CAL in each cluster. We subsequently calculated the cutoff values for the continuous variables of these predictors using the Youden index. In Cluster 1, by building the multifactor logistic regression model, we identified higher PLT and the presence of Symptoms of limb as independent risk factors for predicting concurrent CAL (Figure 2A), with the optimal cutoff value for PLT being 428.5. In Cluster 2, the multifactor logistic regression model revealed that higher PLT, higher CRP, higher TBIL, longer IVIG days, male gender, and the presence of Symptoms of limb acted as independent risk factors for predicting concurrent CAL (Figure 2B). The optimal cutoff values were 453 for PLT, 156.42 for CRP, 5.95 for TBIL, and 8.5 for IVIG days. In Cluster 3, we found that higher PLT, higher CRP, higher WBC, and longer IVIG days served as independent risk factors for predicting concurrent CAL (Figure 2C), with the optimal cutoff values being 405.5 for PLT, 142.34 for CRP, 14.815 for WBC, and 8.5 for IVIG days. We visualized the predictive models for the three clusters using a nomogram (Figures 2D–F).

### 3.4 Model evaluation and validation for three clusters

**Figure 3:**
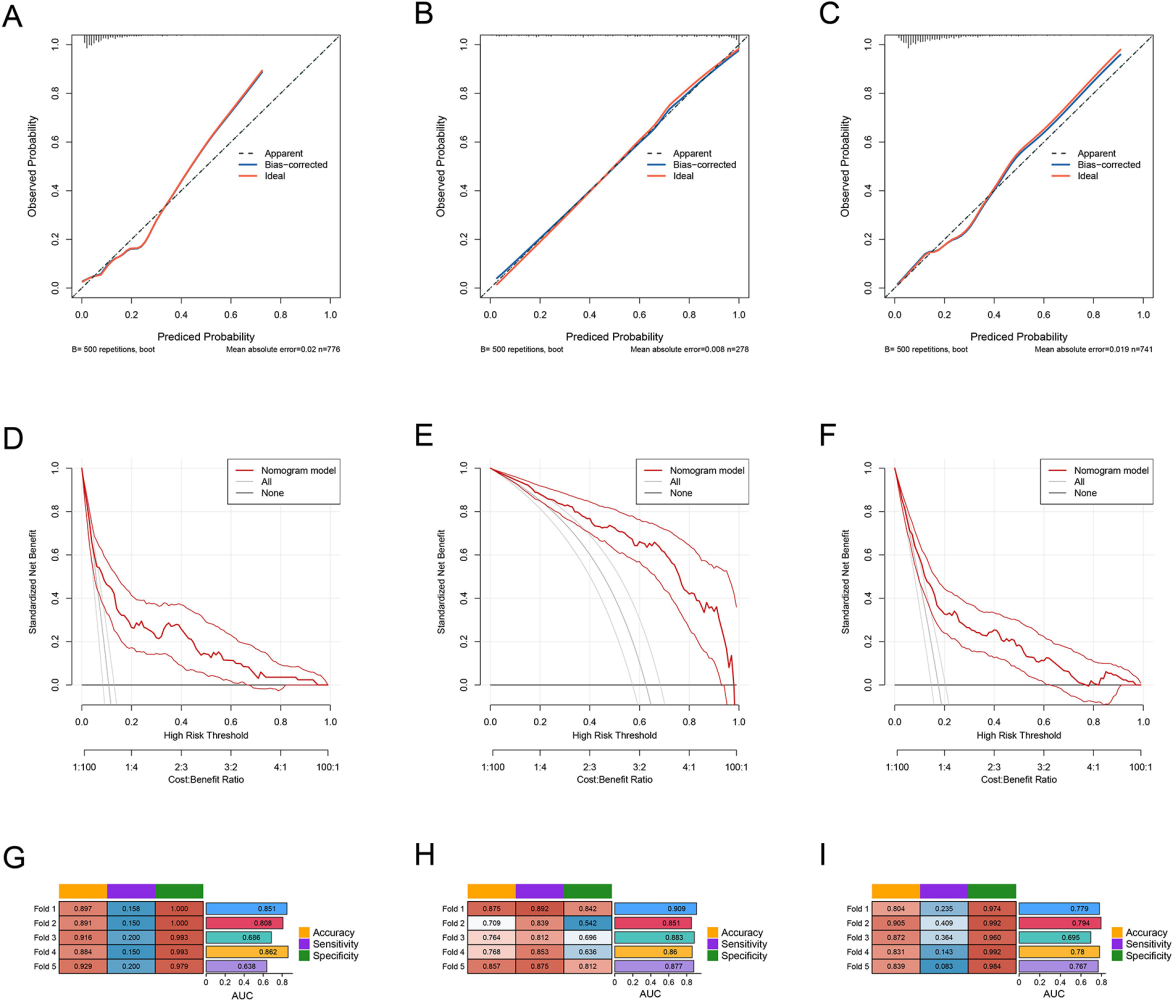
**(A**–**C)** the ROC curves of PLT, CRP, and IVIG days in the three subgroups: cluster 1, cluster 2, and cluster 3. **(D)** The ROC curves of the predictive model in the overall sample set and the three subgroups: cluster 1, cluster 2, and cluster 3.

To evaluate the performance of the predictive models for each cluster, we first constructed the ROC curves for the models of the three clusters. The AUC for Cluster 1 was 0.789 (95% CI: 0.732–0.845), the AUC for Cluster 2 was 0.894 (95% CI: 0.856–0.932), and the AUC for Cluster 3 was 0.773 (95% CI: 0.727–0.819) (Figures 2G–I). We also constructed the calibration curves (Figures 3A–C) and DCA curves (Figures 3D–F) for the models of the three clusters. The results showed that the predictive accuracy and efficacy of the models for all three clusters were satisfactory. Finally, through five-fold cross-validation, we further confirmed that the models for the three clusters exhibited good predictive performance (Figures 3G–I).

## 4 Discussion

This study conducted a clustering analysis of KD patients, revealing the association between typical signs and routine blood parameters and the future occurrence of CAL in children. The main findings were: (1) KD patients exhibited significant clustering based on typical signs and routine blood parameters; (2) higher PLT, elevated CRP, and prolonged IVIG days were independent risk factors for CAL across different subgroups; (3) the predictive model constructed from PLT, CRP, and IVIG days demonstrated good predictive performance in various subgroups.

Although KD is commonly recognized as a single disease entity, its impact on small and medium-sized blood vessels throughout the body affects multiple major organs, leading to diverse clinical features and significant heterogeneity in outcomes (3, 14, 15). Our study conducted a clustering analysis of KD patients and identified three subgroups with distinct clustering characteristics. In Cluster 1, overall inflammatory markers were low, with CRP, ESR, and WBC at their lowest levels, and the patient is the youngest. This indicates that these patients exhibit a low inflammatory response, making them less likely to experience vascular damage and having a lower probability of developing CAL (16). Cluster 2 showed a significantly higher proportion of incomplete Kawasaki disease (IKD) compared to the other two clusters. Since the current understanding of KD relies on typical signs for diagnosis, the manifestations in IKD patients may not be fully expressed. This often leads to prolonged disease duration and delayed treatment (17). As a result, the lack of timely suppression of inflammation greatly increases the risk of IVIG resistance and CAL (18, 19). Additionally, these patients exhibited significantly elevated PLT levels, which may be attributed not only to the absence of timely anti-PLT treatment but also to the delayed increase in PLT associated with KD (20). Cluster 3 primarily presented with liver involvement, characterized by significantly elevated ALT, GGT, and TBIL levels. Although some studies have indicated that these markers are independent risk factors for CAL (21, 22), they more often suggest a higher likelihood of IVIG resistance (23), consistent with our findings. Prior to our study, Hao Wang et al. first identified the high clustering nature of KD and found that patients in the high inflammatory group had a greater incidence of CAL; those with liver involvement had a higher probability of IVIG resistance and a greater incidence of CAL; and patients with fewer typical signs were more likely to develop CAL (13).

During the acute phase of KD, inflammation primarily targets small and medium-sized blood vessels, affecting multiple organs and systems. Among these, CAL resulting from coronary artery involvement represent the most common and significant complication of KD. CAL has also become the leading cause of acquired heart disease in children in developed countries (3). Our study identified PLT, CRP, and the duration of IVIG treatment as consistent independent risk factors across various subgroups. CRP, as the most common inflammatory marker, rises rapidly in response to inflammation or infection due to cellular damage that triggers the release of cytokines and interleukins, stimulating the liver to synthesize and release CRP (16, 24). Elevated CRP levels in KD often indicate more severe vascular inflammation, increasing the likelihood of coronary artery damage. Previous studies have also found that children with CAL had significantly higher CRP levels compared to those without CA (25–28). Regarding PLT, earlier research has shown that patients with higher PLT counts are more likely to develop CAL than those with normal or mildly elevated counts. Increased PLT has been identified as an independent risk factor for CAL in KD. This may be due to the vascular inflammation and damage in KD patients, which leads to PLT aggregation at sites of inflammation and injury (20). When the coronary arteries become inflamed, PLTs aggregate significantly, releasing pro-inflammatory mediators such as PLT factors, inflammatory cytokines, and adhesion molecules. The extensive activation of PLTs may also result in functional abnormalities, making them more likely to adhere to damaged endothelial cells, perpetuating and exacerbating the vascular inflammatory response, ultimately increasing the incidence of CAL (29, 30). IVIG, the first-line treatment for KD, significantly reduces the occurrence of CAL; however, a small proportion of patients still develop CAL. Current research indicates that the timing, duration, and dosage of IVIG administration are closely related to the occurrence and duration of CAL (3). In our study, the AUC value for PLT was highest in Cluster 2, which notably also had the longest duration of fever and IVIG treatment. This suggests that one reason for elevated PLT levels may be their delayed increase. Early in the course of KD, PLT counts are typically within normal ranges or only slightly elevated. However, as the disease progresses, particularly during the second to third week of illness, PLT counts often rise significantly. Children with CAL face an ongoing risk of elevated PLT counts even in the later stages of the disease (20).

We recognize the potential differences between Kawasaki disease patients and the heterogeneity of individual outcomes. Through cluster analysis, we have confirmed the importance of identifying different subgroups within Kawasaki disease patients, ultimately categorizing them into three typical clusters. Among these clusters, we found significant differences in the outcomes of CAL. For patients resembling cluster 1, they are usually younger and exhibit less significant increases in inflammatory markers such as WBC and CRP. Therefore, we should actively utilize the first nomogram for predicting CAL. In the case of patients similar to cluster 2, they often lack obvious clinical signs, which may lead to a delay in diagnosis. When the duration of illness exceeds 8.5 days, even in the absence of typical Kawasaki disease manifestations, we should still consider the combined use of IVIG and aspirin for treatment, especially when PLT exceeds 453, CRP exceeds 156.42, or TBIL is greater than 5.95. Timely treatment and follow-up echocardiograms are essential to monitor the condition of the coronary arteries. At the same time, patients similar to cluster 3 primarily present with liver dysfunction. Elevated levels of ALT, AST, and TBIL suggest that the inflammation may be quite severe. In this case, we should actively conduct liver protection treatments and use the third nomogram to predict CAL. This approach not only aids in managing coronary artery health but also helps assess whether high-risk patients without CAL require appropriate reduction in aspirin dosage or adjustments to their treatment regimen. Moreover, the subgroup of patients with liver dysfunction typically exhibits higher IVIG resistance, which significantly impacts the occurrence of CAL. Thus, after the initial use of IVIG, we need to closely monitor the patient's temperature changes and clinical manifestations, and promptly devise the next treatment plan in the event of IVIG resistance. This is crucial for the prevention of CAL.

However, our study also has limitations. As a retrospective study, biases are inevitable due to constraints in data completeness, accuracy, and sample size. Additionally, our data was validated only in a single center, lacking multi-center validation across different populations and regions. We also recognize the need to include more valuable variables beyond demographic characteristics, clinical manifestations, and routine laboratory indicators. Future research should consider early imaging markers, genomic, transcriptomic, and proteomic features, as well as additional demographic and socio-environmental factors, to help establish a more accurate and comprehensive predictive model. In the future, we aim to conduct more comprehensive, prospective, large-sample, multi-center studies to explore additional variables and subgroups, thereby aiding in the precise diagnosis and treatment of KD.

## 5 Conclusions

In summary, our study identified three clusters among Kawasaki disease patients and developed predictive models for complications of CAL for each cluster. This provides clinicians with a more robust theoretical foundation for predicting CAL in Kawasaki disease, thereby enhancing the precision of individualized treatment for Kawasaki disease patients.

## Data Availability

The original contributions presented in the study are included in the article/Supplementary Material, further inquiries can be directed to the corresponding authors.
