# Supplementary material for: A universal model for predicting coronary artery lesions in subgroups of kawasaki disease in China: based on cluster analysis
Source: Front Cardiovasc Med. 2025 Mar 12;12:1532768. doi: 10.3389/fcvm.2025.1532768 (PMC11936964; doi:10.3389/fcvm.2025.1532768)
Supplement: Supplementary file 1 [file Table1.docx]

**s1: Demographic features, clinical presentation, and clinical laboratory test results of the total cohort and each subgroup**

| Factor | Total(n=1795) | Cluster1(n=776) | Cluster2(n=278) | Cluster3(n=741) | p |
| --- | --- | --- | --- | --- | --- |
| Age | 2.62±2.01 | 1.73±1.27 | 2.34±1.96 | 3.66±2.16 | 0.004 |
| HB | 113(105-121) | 112(104-119) | 105(97-113) | 117(110-124) | <0.001 |
| PLT | 359.24±127.04 | 335.14±86.32 | 511.58±179.34 | 327.33±94.54 | <0.001 |
| WBC | 14.19±5.37 | 12.22±4.36 | 17.89±6.3 | 14.87±5.03 | <0.001 |
| N | 61.49±17.38 | 50.36±15.4 | 59.36±14.49 | 73.95±10.9 | <0.001 |
| L | 28.81±14.82 | 38.47±13.32 | 29.86±12.94 | 18.29±8.67 | <0.001 |
| ESR | 60.92±28 | 52.78±26.47 | 74.41±29.22 | 64.39±26.41 | 0.014 |
| CRP | 77.6±50.42 | 57.59±41.69 | 87.71±53.65 | 94.76±50.11 | <0.001 |
| ALT | 56(15-67) | 38(13-46) | 41(14-49) | 81(17-112) | <0.001 |
| GGT | 69(16-94) | 50(14-66) | 63(19-78) | 91(18-140) | <0.001 |
| TBIL | 15(6.5-17) | 11(5.7-11) | 12(5.82-11) | 21(8.4-32) | <0.001 |
| Fever days | 5.65±2.13 | 5.24±1.7 | 8.12±2.83 | 5.14±1.49 | <0.001 |
| IVIG days | 6.17±1.85 | 5.83±1.25 | 8.37±2.82 | 5.69±1.23 | 0.037 |
| Sex |  |  |  |  | 0.011 |
| Female | 665(37.05) | 289(37.24) | 82(29.5) | 294(39.68) |  |
| Male | 1130(62.95) | 487(62.76) | 196(70.5) | 447(60.32) |  |
| Ethic |  |  |  |  | <0.001 |
| Han ethnicity | 1524(84.9) | 696(89.69) | 212(76.26) | 616(83.13) |  |
| Ethnic minorities | 271(15.1) | 80(10.31) | 66(23.74) | 125(16.87) |  |
| Oral mucosal involvement |  |  |  |  | <0.001 |
| No | 326(18.16) | 111(14.3) | 113(40.65) | 102(13.77) |  |
| Yes | 1469(81.84) | 665(85.7) | 165(59.35) | 639(86.23) |  |
| Conjunctival injection |  |  |  |  | <0.001 |
| No | 247(13.76) | 96(12.37) | 101(36.33) | 50(6.75) |  |
| Yes | 1548(86.24) | 680(87.63) | 177(63.67) | 691(93.25) |  |
| Rash |  |  |  |  | <0.001 |
| No | 501(27.91) | 182(23.45) | 167(60.07) | 152(20.51) |  |
| Yes | 1294(72.09) | 594(76.55) | 111(39.93) | 589(79.49) |  |
| Cervical Lymphadenopathy |  |  |  |  | <0.001 |
| No | 846(47.13) | 516(66.49) | 167(60.07) | 163(22) |  |
| Yes | 949(52.87) | 260(33.51) | 111(39.93) | 578(78) |  |
| Symptoms of limb |  |  |  |  | <0.001 |
| No | 936(52.14) | 546(70.36) | 141(50.72) | 249(33.6) |  |
| Yes | 859(47.86) | 230(29.64) | 137(49.28) | 492(66.4) |  |
| IKD |  |  |  |  | <0.001 |
| No | 1235(68.8) | 528(68.04) | 134(48.2) | 573(77.33) |  |
| Yes | 560(31.2) | 248(31.96) | 144(51.8) | 168(22.67) |  |
| CAL |  |  |  |  | <0.001 |
| No | 1407(78.38) | 692(89.18) | 104(37.41) | 611(82.46) |  |
| Yes | 388(21.62) | 84(10.82) | 174(62.59) | 130(17.54) |  |
| IVIG resistance |  |  |  |  | <0.001 |
| No | 1642(91.48) | 753(97.04) | 231(83.09) | 658(88.8) |  |
| Yes | 153(8.52) | 23(2.96) | 47(16.91) | 83(11.2) |  |
