# Supplementary material for: A universal model for predicting coronary artery lesions in subgroups of kawasaki disease in China: based on cluster analysis
Source: Front Cardiovasc Med. 2025 Mar 12;12:1532768. doi: 10.3389/fcvm.2025.1532768 (PMC11936964; doi:10.3389/fcvm.2025.1532768)
Supplement: Supplementary file 2 [file Table2.docx]

**S2: Comparison of clinical features between concurrent CAL groups in Cluster 1.**

| Factor | CAL(n=84) | nCAL(n=692) | *p* |
| --- | --- | --- | --- |
| Age | 1.57±1.65 | 1.75±1.22 | 0.332 |
| HB | 109.51±10.41 | 111.89±11.62 | 0.053 |
| PLT | 414.98±108.27 | 325.45±77.97 | <0.001 |
| WBC | 13.82±4.73 | 12.03±4.28 | 0.001 |
| N | 49(41.3-59) | 51(41.58-62) | <0.001 |
| L | 39(29.77-46) | 38(28.7-46) | <0.001 |
| ESR | 50(31-66) | 53(36-71) | <0.001 |
| CRP | 69.52±44.78 | 56.14±41.1 | 0.01 |
| ALT | 36(13-44) | 39(14-46) | 0.75 |
| GGT | 52(13.75-71) | 49(14-64) | 0.003 |
| TBIL | 11.94±10.15 | 10.47±8.52 | 0.207 |
| Fever days | 5.48±1.66 | 5.22±1.7 | 0.18 |
| IVIG days | 6.2±1.31 | 5.79±1.23 | 0.007 |
| Sex |  |  | 0.105 |
| Female | 24(28.57) | 265(38.29) |  |
| Male | 60(71.43) | 427(61.71) |  |
| Ethic |  |  | 0.952 |
| Han ethnicity | 76(90.48) | 620(89.6) |  |
| Ethnic minorities | 8(9.52) | 72(10.4) |  |
| Oral mucosal involvement |  |  | 1 |
| No | 12(14.29) | 99(14.31) |  |
| Yes | 72(85.71) | 593(85.69) |  |
| Conjunctival injection |  |  | 0.507 |
| No | 8(9.52) | 88(12.72) |  |
| Yes | 76(90.48) | 604(87.28) |  |
| Rash |  |  | 0.624 |
| No | 22(26.19) | 160(23.12) |  |
| Yes | 62(73.81) | 532(76.88) |  |
| Cervical lymphadenopathy |  |  | 0.167 |
| No | 62(73.81) | 454(65.61) |  |
| Yes | 22(26.19) | 238(34.39) |  |
| Symptoms of limb |  |  | <0.001 |
| No | 35(41.67) | 511(73.84) |  |
| Yes | 49(58.33) | 181(26.16) |  |
