# Supplementary material for: A universal model for predicting coronary artery lesions in subgroups of kawasaki disease in China: based on cluster analysis
Source: Front Cardiovasc Med. 2025 Mar 12;12:1532768. doi: 10.3389/fcvm.2025.1532768 (PMC11936964; doi:10.3389/fcvm.2025.1532768)
Supplement: Supplementary file 3 [file Table3.docx]

**S3: Comparison of clinical features between concurrent CAL groups in Cluster 2.**

| Factor | CAL(n=174) | nCAL(n=104) | *p* |
| --- | --- | --- | --- |
| Age | 2.26±2.16 | 2.46±1.58 | 0.37 |
| HB | 104(96-113) | 106(97.75-114) | 0.698 |
| PLT | 577.32±181.50 | 401.6±107.96 | <0.001 |
| WBC | 19(13-22) | 17(12.6-21) | <0.001 |
| N | 57.32±15.09 | 62.77±12.79 | 0.001 |
| L | 31.19±13.58 | 27.63±11.53 | 0.021 |
| ESR | 72(48.25-94) | 79(60-98) | <0.001 |
| CRP | 94.86±57.25 | 75.73±44.78 | 0.002 |
| ALT | 45(15-52) | 36(12-48) | 0.75 |
| GGT | 70(24-86) | 51(15-56) | 0.003 |
| TBIL | 12.62±10.98 | 10.29±7.32 | 0.035 |
| Fever days | 8.44±2.99 | 7.59±2.48 | 0.011 |
| IVIG days | 8.89±3.08 | 7.52±2.07 | <0.001 |
| Sex |  |  | 0.001 |
| Female | 39(22.41) | 43(41.35) |  |
| Male | 135(77.59) | 61(58.65) |  |
| Ethic |  |  | 0.413 |
| Han ethnicity | 136(78.16) | 76(73.08) |  |
| Ethnic minorities | 38(21.84) | 28(26.92) |  |
| Oral mucosal involvement |  |  | 0.416 |
| No | 67(38.51) | 46(44.23) |  |
| Yes | 107(61.49) | 58(55.77) |  |
| Conjunctival injection |  |  | 0.025 |
| No | 54(31.03) | 47(45.19) |  |
| Yes | 120(68.97) | 57(54.81) |  |
| Rash |  |  | 0.075 |
| No | 97(55.75) | 70(67.31) |  |
| Yes | 77(44.25) | 34(32.69) |  |
| Cervical lymphadenopathy |  |  | 0.208 |
| No | 110(63.22) | 57(54.81) |  |
| Yes | 64(36.78) | 47(45.19) |  |
| Symptoms of limb |  |  | <0.001 |
| No | 66(37.93) | 75(72.12) |  |
| Yes | 108(62.07) | 29(27.88) |  |
