# Supplementary material for: A universal model for predicting coronary artery lesions in subgroups of kawasaki disease in China: based on cluster analysis
Source: Front Cardiovasc Med. 2025 Mar 12;12:1532768. doi: 10.3389/fcvm.2025.1532768 (PMC11936964; doi:10.3389/fcvm.2025.1532768)
Supplement: Supplementary file 4 [file Table4.docx]

**S4: Comparison of clinical features between concurrent CAL groups in Cluster 3.**

| Factor | CAL(n=130) | nCAL(n=611) | *p* |
| --- | --- | --- | --- |
| Age | 4.01±2.68 | 3.59±2.03 | 0.099 |
| HB | 115(106.25-120) | 117(110-125) | 0.698 |
| PLT | 370.93±108.77 | 318.06±88.6 | <0.001 |
| WBC | 18(13.85-21) | 14(11.32-17) | <0.001 |
| N | 74.62±10.88 | 73.8±10.91 | 0.435 |
| L | 18±8.8 | 18.35±8.65 | 0.673 |
| ESR | 65(50.5-86) | 64(47-82) | <0.001 |
| CRP | 121.84±54.14 | 89±47.29 | <0.001 |
| ALT | 85(21-121) | 80(16-109) | 0.75 |
| GGT | 108(21.4-184) | 88(18-132) | 0.003 |
| TBIL | 23.53±18.99 | 20.45±17.95 | 0.092 |
| Fever days | 5.56±1.52 | 5.05±1.47 | <0.001 |
| IVIG days | 6.18±1.53 | 5.58±1.13 | <0.001 |
| Sex |  |  | 0.047 |
| Female | 41(31.54) | 253(41.41) |  |
| Male | 89(68.46) | 358(58.59) |  |
| Ethic |  |  | 0.097 |
| Han ethnicity | 115(88.46) | 501(82) |  |
| Ethnic minorities | 15(11.54) | 110(18) |  |
| Oral mucosal involvement |  |  | 0.999 |
| No | 18(13.85) | 84(13.75) |  |
| Yes | 112(86.15) | 527(86.25) |  |
| Conjunctival injection |  |  | 0.042 |
| No | 3(2.31) | 47(7.69) |  |
| Yes | 127(97.69) | 564(92.31) |  |
| Rash |  |  | 0.999 |
| No | 27(20.77) | 125(20.46) |  |
| Yes | 103(79.23) | 486(79.54) |  |
| Cervical lymphadenopathy |  |  | 0.006 |
| No | 41(31.54) | 122(19.97) |  |
| Yes | 89(68.46) | 489(80.03) |  |
| Symptoms of limb |  |  | 0.999 |
| No | 44(33.85) | 205(33.55) |  |
| Yes | 86(66.15) | 406(66.45) |  |
