# Supplementary material for: A universal model for predicting coronary artery lesions in subgroups of kawasaki disease in China: based on cluster analysis
Source: Front Cardiovasc Med. 2025 Mar 12;12:1532768. doi: 10.3389/fcvm.2025.1532768 (PMC11936964; doi:10.3389/fcvm.2025.1532768)
Supplement: Supplementary file 5 [file Table5.docx]

**S4: Five-fold cross-validation of the predictive model in the overall sample set and three clusters.**

|  | AUC | Accuracy | Sensitivity | Specificity |
| --- | --- | --- | --- | --- |
| Total |  |  |  |  |
| No.1 | 0.832 | 0.836 | 0.38 | 0.948 |
| No.2 | 0.784 | 0.844 | 0.38 | 0.975 |
| No.3 | 0.874 | 0.864 | 0.524 | 0.967 |
| No.4 | 0.841 | 0.864 | 0.487 | 0.968 |
| No.5 | 0.845 | 0.877 | 0.5 | 0.979 |
| Cluster1 |  |  |  |  |
| No.1 | 0.834 | 0.919 | 0.333 | 1 |
| No.2 | 0.848 | 0.912 | 0.154 | 0.985 |
| No.3 | 0.837 | 0.932 | 0.533 | 0.977 |
| No.4 | 0.849 | 0.919 | 0.267 | 0.993 |
| No.5 | 0.797 | 0.906 | 0.263 | 1 |
| Cluster2 |  |  |  |  |
| No.1 | 0.89 | 0.819 | 0.781 | 0.85 |
| No.2 | 0.824 | 0.736 | 0.636 | 0.821 |
| No.3 | 0.809 | 0.806 | 0.727 | 0.872 |
| No.4 | 0.821 | 0.75 | 0.762 | 0.733 |
| No.5 | 0.845 | 0.778 | 0.683 | 0.903 |
| Cluster3 |  |  |  |  |
| No.1 | 0.731 | 0.848 | 0.28 | 0.973 |
| No.2 | 0.812 | 0.862 | 0.273 | 0.974 |
| No.3 | 0.733 | 0.755 | 0.135 | 0.98 |
| No.4 | 0.719 | 0.835 | 0.115 | 1 |
| No.5 | 0.735 | 0.885 | 0.235 | 0.975 |
